# Preliminary evidence that hydrostatic edema may contribute to the formation of diffuse alveolar damage in a Holstein calf model

**DOI:** 10.12688/f1000research.14153.1

**Published:** 2018-03-26

**Authors:** Joseph M. Neary, Dee Church

**Affiliations:** 1 Departments of Animal and Food Sciences, College of Agricultural Sciences and Natural Resources, Texas Tech University, Lubbock, TX, 79409, USA

**Keywords:** acute respiratory distress syndrome, pneumonia, congestive heart failure, hypertension, left ventricle

## Abstract

**Background:** Two notable findings of clinically healthy feedlot cattle suggest they may have pulmonary hydrostatic edema during the finishing phase of production: increased pulmonary arterial wedge pressures and pulmonary venous hypertrophy. The goal of this study was to determine if increased pulmonary arterial wedge pressure (PAWP) in a Holstein calf could lead to diffuse alveolar damage consistent with the early, exudative phase of acute interstitial pneumonia of feedlot cattle.

**Methods:** Six male Holstein dairy calves were given daily subcutaneous injections of the nonspecific ß-adrenergic agonist isoprenaline (10 mg/kg/d), to induce left ventricular diastolic dysfunction, or sterile water for 14 days. On Day 14, pulmonary arterial pressures and wedge pressures were measured, echocardiography performed, and the ratio of mitral valve flow velocity (E) to septal lengthening velocity (e’) calculated. Calves were euthanized on Day 15 and lung lesions semi-quantitatively scored.

**Results:** Mean PAWP was 12 ± 1 mm Hg in calves that received isoprenaline and 7 ± 1 mm Hg in controls ( *P = *0.01). Calves that received isoprenaline tended to have greater relative wall thickness than control calves ( *P = *0.15) and greater E/e’ ratios ( *P = *0.16), suggestive of concentric hypertrophy and diastolic dysfunction, respectively. Calves that received isoprenaline also tended to have a left ventricle and interventricular septum that was 29 ± 10 g heavier than control calves ( *P = *0.10) when controlling for body mass. Hyaline membranes, the hallmark feature of diffuse alveolar damage, were evident in lung sections from all calves that received isoprenaline but none of the controls.

**Conclusions:** Consistent with prior pathological and physiological studies of feedlot cattle, this study provides preliminary evidence that cattle presenting with clinical signs and pathology consistent with early stage acute interstitial pneumonia could be attributable to hydrostatic edema associated with left ventricular failure.

## Introduction

Feedlot cattle are susceptible to two diseases of the cardiopulmonary system as they approach slaughter weight: congestive heart failure ^1^ and acute interstitial pneumonia (AIP) ^2^. In human medicine, and for the purpose of our study, acute interstitial pneumonia was defined as an acute respiratory distress syndrome (ARDS) of unknown etiology that is not primarily attributable to hydrostatic edema due to congestive left ventricular failure, pulmonary venous hypertension, or fluid overload ^3^. In veterinary medicine, a consensus definition of ARDS has been proposed ^4^. Diagnosing feedlot cattle using the proposed criteria, however, is not feasible largely because the diagnostic tools for ruling out hydrostatic edema are not available; consequently, it has been said that definitive diagnosis of AIP in cattle requires histopathologic evaluation of lung tissue obtained postmortem ^5^. The histological counterpart of ARDS is typically diffuse alveolar damage (DAD), defined by the presence of hyaline membranes ^6^.

If an animal dies in the acute, exudate phase of AIP, then hyaline membranes and hemorrhage may be the only lesions observed ^5^. This is problematic because these lesions are not pathognomonic for ARDS and may be attributable to hydrostatic edema. Furthermore, there are two notable findings of clinically healthy feedlot cattle that suggest they may be at risk of congestive left heart failure, and therefore pulmonary hydrostatic edema, during the finishing phase: increased pulmonary arterial wedge pressures (PAWP) and pulmonary venous hypertrophy ^7^. This leads us to speculate whether cattle presenting with clinical signs and pathology consistent with early stage AIP could, at least in some instances, be attributable to hydrostatic edema associated with left ventricular failure. The goal of this study was to determine if increased PAWP due to left ventricular dysfunction in a Holstein calf model could lead to pulmonary lesions consistent with the early, exudative phase of AIP in feedlot cattle.

## Methods

### Overview

Six, day-old male, intact clinically healthy Holstein dairy calves were collected from a farm in West Texas and individually housed under normoxic conditions (altitude: 975 m). At 7-days of age (Day 1 of the study), calves (n = 3) were given daily subcutaneous injections of the nonspecific ß-adrenergic agonist isoprenaline (10 mg/kg/d) (henceforth, ISO calves) or an equivalent volume of sterile water (controls, n = 3) for 14 days. On Day 14, pulmonary arterial pressures and wedge pressures were measured and echocardiography performed. Calves were euthanized on Day 15. The heart and lungs were weighed, and lung sections histologically evaluated.

### Ethical considerations

 Institutional Animal Care and Use Committee approval was granted prior to initiation of the study (Protocol 17013-02). Efforts were made to ameliorate animal suffering by following recommendations for the housing and care of animals provided by the National Research Council ^8^.

### Study site, housing, and feed

Day-old male, intact, male Holstein calves were obtained from one commercial dairy in West Texas. Six calves were included in this preliminary study as, to our knowledge, no similar studies had been performed in this species. Calves were fed 4 to 5 L of colostrum within 12 hours of birth. They were given 3 L of non-medicated milk replacer (22% crude protein, 15% crude fat) twice per day throughout the study (Purina® Herd Maker®, Gray Summit, MO, USA) and had ad libitum access to water. From 1 week of age (Day 1) calves had *ad libitum* access to a calf starter (Purina®, Ampli-Calf® Starter 22, Gray Summit, MO, USA) with 22% crude protein (dry matter basis).

All calves were individually housed in pens with dimensions 1.8 m by 2.3 m (Agri-Plastics, Grassie, ON, Canada). Four calves were housed on a raised slatted floor inside temperature-controlled chambers (temperature 17 ± 3°C). Calves were housed according to body mass so that the heaviest calves at the start of the study (one control and one experimental calf) were housed in one chamber (Calves 4 and 5) and the lightest calves (Calves 1 and 2) in another chamber. One control (Calf 3) and one experimental calf (Calf 6), were housed in shaded outdoor pens with straw bedding on a sloped concrete floor. The pens were moved to new locations every 3 days and the inside of the pens cleaned with disinfectant (Virkon S, DuPont, Wilmington, DE, USA). Soiled straw was removed once daily, and all straw was replaced every 3 days.

### Isoprenaline/Isoproterenol

To our knowledge, isoprenaline (DL-Isoproterenol hydrochloride, 98%, Alfa Aesar, Ward Hill, MA, USA) has not been used in large animal models of heart failure with preserved ejection fraction (HFpEF). The dosage used in this study (10 mg/kg sid) was double the dosage used in a study of equivalent duration in a mouse model ^9^. The isoprenaline was dissolved in sterile water (50 mg/mL) at room temperature prior to subcutaneous injection in the neck. Alternating sides of the neck were used. Controls were injected subcutaneously in the neck with an equivalent volume of sterile water (0.2 mL/kg). Treatments were given between 8:30 am and 9:30 am after all calves had been fed milk replacer. Injections were given whilst calves were manually restrained within their pen.

### Pulmonary arterial pressure measurement

Pulmonary arterial pressure (PAP) testing was performed on Day 14. Calves were restrained in a calf chute and a halter used to hold the calf’s head to one side so that the right jugular grove was exposed. The neck was clipped and cleaned with chlorhexidine solution. A 7-French peel-away introducer (IS-07AS, Vascor Medical Corporation, Tarpon Springs, FL, USA) was placed in the jugular vein prior to inserted of a 110 cm, 7 French, polyurethane, modified J-tip wedge pressure catheter (172-110P, Vascor Medical Corporation, Tarpon Springs, FL, USA). A pressure transducer (TranStar DPT, Smiths Medical ASD, Inc., Dublin, OH, USA) was interposed between the catheter to the data acquisition system (IX-TA-220, iWorx Systems, Inc., Dover, NH, USA). The pressure waveforms were recorded and analyzed offline (LabScribe3, iWorx Systems, Inc., Dover, NH, USA). Pressures were analyzed at the end of expiration. The position of the catheter tip was determined by monitoring the change in the pressure waveform as the catheter tip was advanced through the right atrium, right ventricle, and finally into the pulmonary artery.

### Echocardiography

Echocardiography was performed on Day 14. Calves remained standing throughout the procedure. Fractional shortening and ejection fraction (cubed or Teichholz method ^10^) were obtained from right parasternal long-axis views of the left ventricle obtained between intercostal spaces 3 to 6. Relative wall thickness was calculated as two-times the left ventricular free wall diastolic thickness divided by the left ventricular internal diastolic diameter. Early diastolic (e’) septal lengthening velocities and early mitral flow (E) velocities were obtained from a left parasternal apical four-chamber view. The E/e’ ratio is a measure of left ventricular filling pressure and, consequently, a diagnostic measure of diastolic dysfunction ^11^.

### Postmortem examination and histology

Calves were euthanized with intravenous pentobarbital sodium (85 mg/kg) on Day 15 of the study and exsanguinated. Lung lobes were individually weighed. The atria were separated from the ventricles at the atrioventricular junction. The right ventricular free wall (RV) was separated from the left ventricle and septum (LVS). The RV and LVS were individually weighed.

 The left diaphragmatic lobe was perfused with formalin (10%, neutral buffered) at 15 to 20 cm H _2_O for approximately 5 minutes. After 5 days of formalin fixation, lung sections were collected midway along the dorsal aspect of the lobe for histology. Paraffinized sections (4 µm) were stained with hematoxylin and eosin and Masson’s trichrome. Lung sections were semi-quantitatively scored based on severity (0 = no lesion; 1 = mild; 2 = moderate; 3 = severe). The investigator was blinded to the calves’ identities and treatment group. The pulmonary parenchymal lesions scored included interstitial edema, intra-alveolar edema, hemorrhage and hemosiderin, inflammatory infiltrates, type II hyperplasia, and interstitial fibrosis. Pulmonary arterioles (< 500 μm) and veins were scored for medial hypertrophy and adventitial fibrosis. Veins were distinguishable from arteries as they have spiral bundles of muscle that give them a beaded appearance.

### Statistical analyses

Statistical analyses were performed using commercial software ( Stata version 12.1, College Station, TX). Calf 1 was excluded from statistical analyses because a sub-endocardial lesion was detected postmortem that likely affected his cardiac function and, consequently, pulmonary vascular pathophysiology. Between group differences were evaluated using Student’s *t*-test with equal variances. Student’s t-test is a suitable statistical method for small sample sizes (n ≤ 5) even if group sizes are unequal as long as the effect size is expected to be large ^12^. Linear regression was used to determine if there was a significant difference between groups in the mass of the right ventricle, left ventricle and interventricular septum, and lung, while controlling for body mass.

## Results

### Cardiopulmonary pressures

Calves that received ISO had a greater mean PAWP but a lower mean PAP than controls. Mean PAWP was 12 ± 1 mm Hg in the ISO group and 7 ± 1 mm Hg in the control group ( *P =* 0.01). Mean PAP was 23 ± 2 mm Hg in the ISO group and 43.5 ± 8 mm Hg in the control group ( *P =* 0.05). The control group had a greater heart rate (104 ± 9 bpm) than the ISO group (74 ± 4 bpm; *P =* 0.03). Results are presented in Table 1.

**Table 1.** Cardiopulmonary pressures and pulmonary arterial wedge pressures (PAWP) obtained from male intact Holstein calves after 14 days of receiving the nonspecific ß-adrenergic agonist (ß-AA) isoprenaline (10 mg/kg/d) and control calves that received an equivalent volume of sterile water (0.2 mL/kg).

Group	Calf	Right ventricle	Pulmonary artery	Mean PAWP	Heart rate
Control	1 *	51/-12 (19)	30/11 (20)	5	149
	3	65/5 (35)	68/40 (51)	6	95
	5	68/-6 (30)	48/15 (36)	7	112
ß-AA	2	42/1 (12)	39/6 (20)	10	71
	4	43/3 (8)	46/3 (22)	13	70
	6	42/7 (16)	43/19 (27)	12	81

*Left ventricular endocardial lesion identified post-mortem

### Echocardiography

There was no statistical difference in ejection fraction or fractional shortening between the ISO and control groups indicating that left ventricular systolic function was preserved. Ejection fraction was 66 ± 6% in the control calves and 54 ± 6% in the ISO group ( *P =* 0.29). Fractional shortening was 36 ± 4% in the control calves and 29 ± 4% in the ISO group ( *P =* 0.27). ISO calves tended to have greater relative wall thickness than control calves ( *P =* 0.15) and greater E/e’ ratios ( *P =* 0.16) which is suggestive of concentric hypertrophy and diastolic dysfunction, respectively. Results are presented in Table 2.

**Table 2.** Echocardiographic measures of systolic (ejection fraction and fractional shortening) and diastolic (E/e’ ratio) function and left ventricular wall thickness relative to internal diastolic diameter obtained from male intact Holstein calves after 14 days of receiving the nonspecific ß-adrenergic agonist (ß-AA) isoprenaline (10 mg/kg/d) and control calves that received an equivalent volume of sterile water (0.2 mL/kg).

Group	Calf	Ejection fraction,%	Fractional shortening,%	Relative wall thickness	E/e’
Control	1 *	60	32	0.55	13.2
	3	61	30	0.34	8.1
	5	60	32	0.37	8.3
ß-AA	2	43	22	0.38	11.2
	4	63	34	0.52	10.0
	6	57	30	0.49	8.7

* Left ventricular endocardial lesion identified post-mortem

### Pathology

**Figure 1.**
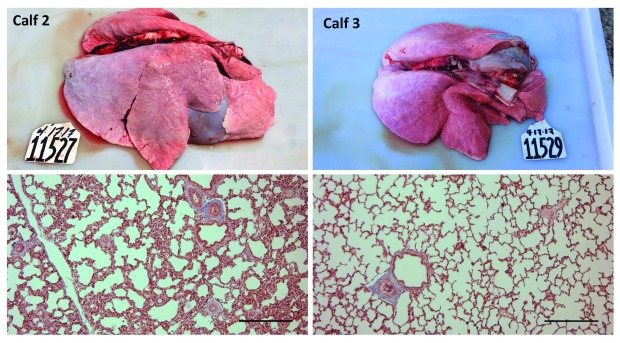
Lungs and pulmonary histology of male intact Holstein calves after 14 days of receiving the nonspecific ß-adrenergic agonist isoprenaline (10 mg/kg/d sid) (Calf 2) or equivalent volume of sterile water (0.2 mL/kg) (Calf 3). Masson’s trichrome. Scale bar = 0.25 mm.

There was no difference in body mass at the end of the study ( *P =* 0.51). The mean body mass was 46.6 kg ± 3.3 kg for the control calves and 42.8 ± 3.4 kg for the ISO group. Calf 1 had endocardial fibrosis extending from the atrio-ventricular boundary to the dorsal aspect of the papillary muscle and approximately 0.5 cm into the myocardium (Supplementary file). Calf 5 had a 1 cm abscess in the right diaphragmatic lobe and Calf 6 had acute fibrinous pneumonia affecting the ventral 20% of the right cranio-ventral lung lobe (Supplementary file). The lungs of Calf 2 appeared hyper-inflated ( Figure 1) and Calf 6 had a mottled icteric liver with rounded margins.

The ISO group tended to have a left ventricle and interventricular septum that was 29 ± 10 g heavier than control calves ( *P =* 0.10) when controlling for body mass. There was no difference between groups in the mass of the lung ( *P =* 0.81) or right ventricle ( *P =* 0.36) when controlling for body mass. Results are presented in Table 3.

**Table 3.** Heart, lung, and body masses of male intact Holstein calves after 14 days of receiving the nonspecific ß-adrenergic agonist (ß-AA) isoprenaline (10 mg/kg/d) and control calves that received equivalent volume of sterile water (0.2 mL/kg).

Group	Calf	Body mass, kg	Left ventricle and septum, g	Right ventricle, g	Lung, g
Control	1 *	32.5	161	69	466
	3	43.3	191	82	650
	5	49.8	208	83	797
ß-AA	2	42.8	207	86	597
	4	48.7	245	90	812
	6	37	174	74	556

* Left ventricular endocardial lesion identified post-mortem

 In general, tissue areas with lesions consistent with DAD were multifocal and often located adjacent to a lobule with a normal healthy appearance. Occasionally, a gradual change from healthy to DAD was observed within the same lobule. All ISO calves had mild or moderate interstitial and intra-alveolar edema, mild or moderate hemorrhage, and type II hyperplasia. Two control calves (Calves 3 and 5) showed mild interstitial edema in some areas. Interstitial inflammatory infiltrates were evident in two calves, particularly Calf 1. No calves showed evidence of interstitial fibrosis. Calves 4 and 6 showed moderate epithelisation of alveoli. Calf 6 also had severe emphysema and honeycomb parenchymal remodeling ( Figure 1). Results are presented in Table 4.

**Table 4.** Pulmonary parenchymal lesions consistent with diffuse alveolar damage in male intact Holstein calves after 14 days of receiving the nonspecific ß-adrenergic agonist (ß-AA) isoprenaline (10 mg/kg/d) and control calves that received equivalent volume of sterile water (0.2 mL/kg).

Group	Calf	Interstitial edema	Hyaline membrane	Hemorrhage	Inflammatory infiltrates	Type II hyperplasia	Fibrosis
Control	1 ^*^	0	0	1	2	1	0
	3	1	0	0	0	0	0
	5	1	0	0	0	0	0
ß-AA	2	2	2	2	1	2	0
	4	2	2	2	0	2	0
	6	2	1	1	0	1	0

*Left ventricular endocardial lesion identified post-mortem

Mild or moderate medial hypertrophy and adventitial fibrosis of pulmonary arterioles was evident in all calves. ISO calves had more substantial medial hypertrophy of the pulmonary veins than controls. Pulmonary veins were not evident in Calf 6. Adventitial fibrosis was not observed except in Calf 4. Results are presented in Table 5.

**Table 5.** Semi-quantitative assessment of medial hypertrophy and adventitial fibrosis in pulmonary arteries (< 500 µm) and veins from male intact Holstein calves after 14 days of receiving the nonspecific ß-adrenergic agonist (ß-AA) isoprenaline (10 mg/kg/d) and control calves that received an equivalent volume of sterile water (0.2 mL/kg).

		Pulmonary arteriole		Pulmonary vein	
Group	Calf	Medial hypertrophy	Adventitial fibrosis	Medial hypertrophy	Adventitial fibrosis
Control	1*	1	2	0	0
	3	1	2	1	0
	5	2	1	1	0
ß-AA	2	1	2	2	0
	4	1	2	2	2
	6	1	1	-	-

## Discussion

The findings of this study indicate that left ventricular dysfunction could feasibly contribute to the development of diffuse alveolar damage, the pathologic correlate of acute interstitial pneumonia, an ARDS of feedlot cattle of unknown etiology. Two weeks of daily injections with isoprenaline led to the development of heart failure with preserved ejection fraction (HFpEF); left ventricular systolic function was preserved but diastolic function was reduced. Given that subclinical HFpEF in a Holstein model has many of the pathologic features and physiologic measurements consistent with ARDS ^13^, this preliminary study indicates that the diagnosis of AIP in a feedlot animal can only be considered as tentative if left ventricular dysfunction has not been ruled out as a primary cause.

The PAWP of the ISO calves were significantly greater than controls but substantially less than the PAWP of 26 mm Hg previously reported in feedlot cattle at the end of the feeding period at the altitude of 1,220 m ^7^. Pulmonary arterial wedge pressures are typically less than 15 mm Hg in non-feedlot cattle and other mammals ^14^; therefore, the DAD seen in the ISO calves in this study is likely attributable to the acute doubling of PAWP over a 2-week period. Feedlot cattle may be able to tolerate much greater PAWP if pressures were to rise insidiously throughout the feeding period. There are a variety of adaptations, in addition to pulmonary arterial and venous remodeling ^15^, that may occur in response to rising pulmonary vascular pressures: first, the lymphatic system is recruitable – it is able to increase the rate of lung-water clearance by over 10-fold. ^16, 17^; and second, alveolar fibrosis may occur to reduce alveolar-capillary membrane permeability to water ^18^. The time period over which pulmonary vascular pressure rise is, therefore, likely to be a key determinant of the pulmonary adaptability.

It is also feasible that isoprenaline induced DAD through forward hemodynamic effects. Increased cardiac contractility and pulse pressure may have had deleterious downstream effects on the pulmonary vasculature ^19, 20^ paving the way for increased fluid flux into the pulmonary interstitium. Whether attributable to backward or forward hemodynamic effects, an increase in pulmonary capillary pressure will lead to mechanical injury of the alveolar-capillary membrane leading to increased capillary permeability and impaired gas exchange ^21^. Although acute damage can be reversed, long term blood-gas barrier disruption leads to lung fibrosis, inflammation, impaired alveolar fluid clearance, and muscularization of pulmonary vessels ^21, 22^.

Interestingly, the two calves housed in shaded outdoor pens on straw bedding had the greatest pulmonary arterial pressures within their respective treatment groups. In a prior study, calves housed on straw that developed bloody scours had histologic evidence of DAD and greater pulmonary arterial pressures than calves housed on slatted flooring that did not develop scours ^23^. It was speculated that intestinal disease may contribute to the development of pulmonary disease in cattle ^23^. Calves housed on straw bedding in our study may have been in the incubation phase of intestinal disease.

 Even though there were only 6 calves in this study, the goal of the study, to determine if left ventricular dysfunction could lead to lesions consistent with the early, exudative phase of AIP, was met. For ethical reasons, decompensated heart failure was not induced; therefore, it is not possible to say whether overt left ventricular failure clinically manifests as an ARDS-like event. Human medical reports suggest, however, that it can present as an ARDS which is why the diagnosis of ARDS requires that acute dyspnea attributable to hydrostatic edema must first be ruled out ^3^. Large-scale prospective cohort studies of feedlot cattle are necessary to determine if pulmonary arterial pressures and PAWP are positively associated with risk of respiratory diseases, such as AIP. We speculate that elevated pulmonary vascular hydrostatic pressures may act synergistically with mediators of acute alveolar damage, such as pathogens or airborne irritants, to promote the development and progression of respiratory disease.

## Data availability

All raw data underlying this study is available from Harvard Dataverse under a CC0 Public Domain Dedication

Images of the heart and lungs are available at http://dx.doi.org/10.7910/DVN/HD1GEI ^24^

Pulmonary histology is available at http://dx.doi.org/10.7910/DVN/UR9MAC ^25^

Hepatic histology images are available at http://dx.doi.org/10.7910/DVN/ZBIVAG ^26^

Pulmonary vascular pressure recordings are available at http://dx.doi.org/10.7910/DVN/SKBMWZ ^27^

## References

[1] Neary JM Booker CW Wildman BK: Right-Sided Congestive Heart Failure in North American Feedlot Cattle. *J Vet Intern Med.* 2016;30(1):326–334. 10.1111/jvim.13789 26547263 PMC4913666

[2] Loneragan GH Gould DH Mason GL: Involvement of microbial respiratory pathogens in acute interstitial pneumonia in feedlot cattle. *Am J Vet Res.* 2001;62(10):1519–1524. 10.2460/ajvr.2001.62.1519 11592313

[3] ARDS Definition Task Force, Ranieri VM Rubenfeld GD: Acute respiratory distress syndrome: the Berlin Definition. *JAMA.* 2012;307(23):2526–2533. 10.1001/jama.2012.5669 22797452

[4] Wilkins PA Otto CM Baumgardner JE: Acute lung injury and acute respiratory distress syndromes in veterinary medicine: consensus definitions: The Dorothy Russell Havemeyer Working Group on ALI and ARDS in Veterinary Medicine. *J Vet Emerg Crit Care.* 2007;17(4):333–339. 10.1111/j.1476-4431.2007.00238.x

[5] Woolums AR: Feedlot Acute Interstitial Pneumonia. *Vet Clin North Am Food Anim Pract.* 2015;31(3):381–9, vi. 10.1016/j.cvfa.2015.05.010 26253266

[6] Sweeney RM McAuley DF: Acute respiratory distress syndrome. *Lancet.* 2016;388(10058):2416–2430. 10.1016/S0140-6736(16)00578-X 27133972 PMC7138018

[7] Neary JM: Epidemiological, physiological and genetic risk factors associated with congestive heart failure and mean pulmonary arterial pressure in cattle.Doctoral dissertation, Department of Clinical Sciences, Colorado State University,2014 Reference Source

[8] Institute for Laboratory Animal Research: Guide for the Care and Use of Laboratory Animals.8th Ed.2011. 21595115

[9] Ma X Song Y Chen C: Distinct actions of intermittent and sustained β-adrenoceptor stimulation on cardiac remodeling. *Sci China Life Sci.* 2011;54(6):493–501. 10.1007/s11427-011-4183-9 21706409

[10] Teichholz LE Kreulen T Herman MV: Problems in echocardiographic volume determinations: echocardiographic-angiographic correlations in the presence of absence of asynergy. *Am J Cardiol.* 1976;37(1):7–11. 10.1016/0002-9149(76)90491-4 1244736

[11] Paulus WJ Tschöpe C Sanderson JE: How to diagnose diastolic heart failure: a consensus statement on the diagnosis of heart failure with normal left ventricular ejection fraction by the Heart Failure and Echocardiography Associations of the European Society of Cardiology. *Eur Heart J.* 2007;28(20):2539–2550. 10.1093/eurheartj/ehm037 17428822

[12] de Winter JC: Using the Student ’s *t*-test with extremely small sample sizes. *Pr Assessment, Res Evalutaion.* 2013;18(10):1–12. Reference Source

[13] Matute-Bello G Downey G Moore BB: An official American Thoracic Society workshop report: features and measurements of experimental acute lung injury in animals. *Am J Respir Cell Mol Biol.* 2011;44(5):725–738. 10.1165/rcmb.2009-0210ST 21531958 PMC7328339

[14] Will DH Alexander AF Reeves JT: High altitude-induced pulmonary hypertension in normal cattle. *Circ Res.* 1962;10(2):172–177. 10.1161/01.RES.10.2.172 14007066

[15] Reid LM: The pulmonary circulation: remodeling in growth and disease. The 1978 J. Burns Amberson lecture. *Am Rev Respir Dis.* 1979;119(4):531–546. 443626 10.1164/arrd.1979.119.4.531

[16] Uhley HN Leeds SE Sampson JJ: Role of pulmonary lymphatics in chronic pulmonary edema. *Circ Res.* 1962;11(6):966–70, Accessed June 23, 2017. 10.1161/01.RES.11.6.966 13995239

[17] Datar SA Johnson EG Oishi PE: Altered lymphatics in an ovine model of congenital heart disease with increased pulmonary blood flow. *Am J Physiol Lung Cell Mol Physiol.* 2012;302(6):L530–40. 10.1152/ajplung.00324.2011 22207591 PMC3311527

[18] Townsley MI Fu Z Mathieu-Costello O: Pulmonary microvascular permeability. Responses to high vascular pressure after induction of pacing-induced heart failure in dogs. *Circ Res.* 1995;77(2):317–325, Accessed June 23, 2017. 10.1161/01.RES.77.2.317 7614719

[19] Li M Scott DE Shandas R: High pulsatility flow induces adhesion molecule and cytokine mRNA expression in distal pulmonary artery endothelial cells. *Ann Biomed Eng.* 2009;37(6):1082–1092. 10.1007/s10439-009-9684-3 19340571 PMC3057109

[20] Li M Stenmark KR Shandas R: Effects of pathological flow on pulmonary artery endothelial production of vasoactive mediators and growth factors. *J Vasc Res.* 2009;46(6):561–571. 10.1159/000226224 19571576 PMC3073484

[21] Guazzi M: Alveolar-capillary membrane dysfunction in heart failure: evidence of a pathophysiologic role. *Chest.* 2003;124(3):1090–1102, Accessed August 17, 2017. 10.1378/chest.124.3.1090 12970042

[22] Chen Y Guo H Xu D: Left ventricular failure produces profound lung remodeling and pulmonary hypertension in mice: heart failure causes severe lung disease. *Hypertension.* 2012;59(6):1170–1178. 10.1161/HYPERTENSIONAHA.111.186072 22508832 PMC3402091

[23] Gonyeau K Subbiah S Klein D: Development of pulmonary arterial hypertension and diffuse alveolar damage in 2-month old Holstein dairy calves following an acute episode of bloody scours [version 1; referees: 1 approved, 1 approved with reservations]. *F1000Res.* 2017;6:1826 10.12688/f1000research.12717.1

[24] Neary J: Heart and lungs of Holstein calves. Harvard Dataverse, V1.2018 10.7910/DVN/HD1GEI

[25] Neary J: Pulmonary histology of Holstein calves. Harvard Dataverse, V1.2018 10.7910/DVN/UR9MAC

[26] Neary J: Hepatic histology of Holstein calves. Harvard Dataverse, V1.2018 10.7910/DVN/ZBIVAG

[27] Neary J: Cardiopulmonary pressures in Holstein calves. Harvard Dataverse, V1.2018 10.7910/DVN/SKBMWZ

