# Supplementary material for: Preliminary evidence that hydrostatic edema may contribute to the formation of diffuse alveolar damage in a Holstein calf model
Source: F1000Res. 2018 Mar 26;7:374. [Version 1] doi: 10.12688/f1000research.14153.1 (PMC6263485; doi:10.12688/f1000research.14153.1)
Supplement: Peer review history

# Peer review: Referee response for version 1

**DOI:** 10.5256/f1000research.15397.r37946

**Published:** 2018-11-28

**Authors:** Min Li

**Affiliations:** 1 Developmental Lung Biology, Cardiovascular Pulmonary Research Laboratories, University of Colorado Denver, Denver, CO, USA

In this small pilot study authors tried to determine if increased pulmonary arterial wedge pressure (PAWP) in a Holstein calf could lead to diffuse alveolar damage (DAD), which provide histologic evidence to diagnose acute interstitial pneumonia (AIP). The results showed that left ventricular dysfunction can associate with, or be one of the cause that a feedlot steer has the sign of AIP (hyaline membranes and type II pneumocyte hyperplasia).

 "If applicable, is the statistical analysis and its interpretation appropriate?"

 Due to the small number of animals used in the study, a qualified statistician is suggested to verify the statistical analyses.

 "Are the conclusions drawn adequately supported by the results?"

 More histology images (figure 1) with clear labels of different type of lesions and pathology findings will help to support the findings shown in Table 4 and 5.

 Specific comments:

 1. Methods

 Postmortem exam and histology

 Whether a board certified veterinary pathologist identified and scored the histologic lesions? 

 Results

 More histology images (Figure 1) with clear labels of different type of lesions and pathology findings will help to support the findings shown in Table 4 and 5.

 2. In the introduction or discussion part, I would like authors to refine the novelty of this study. Increased PAWP due to left ventricular dysfunction can lead to  hydrostatic edema, diffuse alveolar damage, all of which are pathologically correlate with acute interstitial pneumonia. This concept is acknowledged in the cattle field and the results from this study confirmed this concept and suggest that in feedlot, ARDS can not be diagnosed without first rule out LV dysfunction.

I have read this submission. I believe that I have an appropriate level of expertise to confirm that it is of an acceptable scientific standard.

# Peer review: Referee response for version 1

**DOI:** 10.5256/f1000research.15397.r37016

**Published:** 2018-10-23

**Authors:** Amelia Woolums

**Affiliations:** 1 Department of Pathobiology and Population Medicine, Mississippi State University, Starkville, MS, USA

"If applicable, is the statistical analysis and its interpretation appropriate?"

 For a small pilot study, the approach to statistical analysis is acceptable. However, in some cases the statistical analysis may have provided results that are misleading. For example: in the Results section, the authors state that there was no difference in body mass between calves in the two groups. How was this tested? If by the Student’s t-test: per the Methods section, the t-test is only appropriate for small samples of unequal size if the effect is expected to be large. But the difference in body mass between the groups was not large, so it’s not convincing that the results of this analysis are accurate. Body mass of the calves is not a particularly important outcome of this study, but if differences are going to be reported, a different statistical test should be used. 

 Similarly, while the ejection fraction between the two groups was not significantly different, the distribution of values for this outcome in the calves in the two groups looks like EF would likely have been significantly lower in treated calves if more animals had been included. That is, the study likely lacked power to identify a true difference in ejection fraction. Thus this model may not actually induce left heart failure with preserved ejection fraction, as the authors contend. 

 "Are the conclusions drawn adequately supported by the results?"

 Generally yes, with the caveat that the conclusions are based on a very small number of animals. The data depicted in Table 4 support the authors’ contention that experimentally-induced left heart dysfunction was associated with lung lesions typical of early acute interstitial pneumonia: hyaline membranes and type II pneumocyte hyperplasia.

 Specific comments:

 Methods

 Postmortem exam and histology

 What were the qualifications of the investigator who identified and scored the histologic lesions? Was that person a board certified veterinary pathologist? 

 Results

 Do the investigators have any idea why the ISO treated calves had lower heart rates than the control calves?

 Discussion

 The writers note: “…this preliminary study indicates that the diagnosis of AIP in a feedlot animal can only be considered as tentative if left ventricular dysfunction has not been ruled out...”

 Strictly speaking, AIP (acute interstitial pneumonia) is a pathologic diagnosis, which can result from a variety of possible primary insults. This study does suggest that acute left heart dysfunction can be associated with the lung lesion consistent with early AIP (hyaline membranes and type II pneumocyte hyperplasia). A histologic diagnosis of AIP can be made based on evidence of diffuse alveolar damage; this work suggests that any cause of acute left heart failure could be one of several possible causes of AIP. 

 This is in contrast to the clinical definition of ARDS which, as the authors note in the introduction, currently requires rule out of respiratory failure solely due to heart failure. However, it is usually not possible in the feedlot to make the measurements necessary to confirm ARDS and rule out acute left heart failure, based on current definitions. This research does support the concept that a feedlot steer or heifer with acute severe dyspnea may be suffering from a lung lesion resulting solely from acute congestive left heart failure, as well as a lung lesion resulting from infection, pneumotoxicity, or other possible causes of AIP.

I have read this submission. I believe that I have an appropriate level of expertise to confirm that it is of an acceptable scientific standard.
